# TBro: visualization and management of *de novo* transcriptomes

**DOI:** 10.1093/database/baw146

**Published:** 2016-12-26

**Authors:** Markus J. Ankenbrand, Lorenz Weber, Dirk Becker, Frank Förster, Felix Bemm

**Affiliations:** 1 Department of Animal Ecology and Tropical Biology, Biocenter, Am Hubland, 97074 Würzburg, Germany; 2 Department of Bioinformatics, Biocenter, Am Hubland, 97074 Würzburg, Germany; 3 Center for Computational and Theoretical Biology, University of Würzburg, 97074 Würzburg, Germany; 4 Institute for Molecular Plant Physiology and Biophysics, University of Würzburg, 97082 Würzburg, Germany; 5 Department Molecular Biology (Detlef Weigel), Max-Planck-Institute for Developmental Biology, 72076 Tübingen, Germany

## Abstract

RNA sequencing (RNA-seq) has become a powerful tool to understand molecular mechanisms and/or developmental programs. It provides a fast, reliable and cost-effective method to access sets of expressed elements in a qualitative and quantitative manner. Especially for non-model organisms and in absence of a reference genome, RNA-seq data is used to reconstruct and quantify transcriptomes at the same time. Even SNPs, InDels, and alternative splicing events are predicted directly from the data without having a reference genome at hand. A key challenge, especially for non-computational personnal, is the management of the resulting datasets, consisting of different data types and formats. Here, we present TBro, a flexible *de novo* transcriptome browser, tackling this challenge. TBro aggregates sequences, their annotation, expression levels as well as differential testing results. It provides an easy-to-use interface to mine the aggregated data and generate publication-ready visualizations. Additionally, it supports users with an intuitive cart system, that helps collecting and analysing biological meaningful sets of transcripts. TBro’s modular architecture allows easy extension of its functionalities in the future. Especially, the integration of new data types such as proteomic quantifications or array-based gene expression data is straightforward. Thus, TBro is a fully featured yet flexible transcriptome browser that supports approaching complex biological questions and enhances collaboration of numerous researchers.

**Database URL**: tbro.carnivorom.com

## Background

RNA sequencing (RNA-seq) provides a fast and cost-effective method to access transcribed genes in a qualitative and quantitative manner (1, 2). Without prior knowledge this technology enables transcript discovery and quantification at the same time (3). In particular, for non-model organisms and in absence of a reference genome, RNA-seq has been proven a successful strategy to elucidate the role of candidate genes in physiological pathways or developmental programs as well as the underlying molecular mechanisms (4–7).

Nowadays, transcriptome assemblers such as Velvet/Oases (8) and Trinity (9, 10) are capable to accurately reconstruct full length transcripts, even for recently duplicated genes or alternative splice isoforms from RNA-seq data. Most assemblers operate over a broad range of expression levels. The assembled sequences are usually organized into hypothetical genes (unigenes) represented by multiple isoforms. Those isoforms are usually searched for candidate coding regions. Their deduced proteins are annotated by employing homology as well as profile based methods such as InterproScan (11) and Mercator (12). Furthermore, reusing the generated RNA-seq data for tools like RSEM (13) or Salmon (14) provide quantification of isoforms and their subordinate unigenes. Quantification results serve as input for differential expression (DE) testing, one of the major applications of RNA-seq. Both DE testing results as well as isoform annotation are subject to Gene Ontology or gene family enrichment analysis with tools like topGO (15) or GAGE (16) on either whole transcriptomes or curated subsets.

In the end, most *de novo* RNA-seq studies result in a multitude of different datasets, including sequences, their annotation, expression levels and DE as well as co-expression testing results. Since most of the datasets contain thousands of entries they remain hard to handle. The vast amount of different data types necessitates the usage of a simple interface, optimally through a web browser, to allow uniform data access also for non-IT personal. Researchers need to refine functional annotations (e.g. unigene/isoform synonyms or descriptions) or flag individual unigenes or isoforms with personal metadata. Additionally, classification of biologically related unigenes or isoforms into functional groups or protein families is often pivotal to help understanding their specific roles and interplay in given pathways and networks. Currently, only a small number of tools and platforms are available that provides these basic functions. Most tools are tailored for genome reference based RNA-seq studies [e.g. Tripal (17, 18), Intermine (19), TraV (20), RNASeqExpressionBrowser (21)] or aim for a specific species [e.g. dbWFA (22)] with Intermine and Tripal the most feature rich and best maintained tools available. Intermine is specifically designed for the integration and analysis of complex biological data sets on top of genome annotations but comes with a higher hardware footprint and a complex backend not ideal for smaller lab environments. Tripal on the other hand, serves as online biological knowledgement system displaying predefined queries and thus making it inflexible for large amounts of different user requests. Only TrinotateWeb (23) provides a unified way to create, organize, and visualize results from de novo transcriptome studies. However, it allows no multi user access, lacks the ability to store user-defined unigene or isoform collections, offers only a very sparse search interface and is not capable to provide pathway information. Beyond that, it is hard to extend since the back-end does neither rely on a documented database schema, such as Chado nor does the front-end make use of a modular web service system necessary for new visualizations or analyses. Here we present TBro, a flexible *de novo* transcriptome browser, written to overcome the above-mentioned constraints thereby enabling researchers to analyse and share their data in a collaborative and standardized manner.

## Features

TBro represents an easy to use multi-user *de novo* transcriptome data mining platform. It is developed as web application, works across platforms, and is browser independent. The TBro interface provides structured access to a given transcriptome and its annotation by modelling unigene → isoform relations. Unigene subpages (e.g. http://tbro.carnivorom.com/tbro/details/byId/439690) offer a tabular list of all available isoforms including high level visualization functions for expression profiles and DE testing results. Similarly, isoforms are presented on individual comprehensive subpages allowing users to inspect annotations and metadata (e.g. synonyms and descriptions) as well as enabling visualization of analysis results (e.g. quantifications or DE testing results) dynamically in one place (e.g. http://tbro.carnivorom.com/details/byId/439692). Isoforms and annotated peptides are sent directly to NCBI’s blast suite (24). Annotated features like repeats, predicted peptides and interpro hits are displayed in an overview graph and listed as separate tables. If available, a link to the underlying external database entry is provided. Simple annotations like Gene Ontology terms, MapMan bins and Enzyme commision numbers are displayed underneath. All coordinate-based annotations (e.g. open reading frames, protein domains) as well as expression profiles and differential expression results are visualized by CanvasXpress (25). The visualization itself as well as the underlying data tables are modified dynamically using the context menu of the CanvasXpress library. Users can simply change graphical parameters, scaling and limits of the plots as well as transform or correlate them in different ways. In addition, users can add a custom alias and description for each unigene or isoform at the top of each subpage. Advanced users can use TBro’s web services as an application programming interface (API) to access and integrate data into other applications.

One of TBro’s major achievements is the implemented cart system to comfortably organize and analyse user-specified collections of unigenes and isoforms. They are compiled from the underlying transcriptome database by different exploration methods. Users can select unigenes or isoforms of interest by homology searches (e.g. BLAST), annotated protein signatures (e.g. Interpro) or pathway assignments (e.g. KEGG) as well as through fine grained filtering of expression and differential expression results. Furthermore, users can search for unigenes and isoforms by their id or alias or enter complete paragraphs of a paper to mine them for potential hits. The search for an id or alias is carried out in a strict mode to perfectly match a database entry or in non-strict mode to expand the results to related entries. The latter is used to easily retrieve all isoforms for a unigene. Resulting hits are further refined by simple string or data type specific filters. Results are usually displayed as tables and selected rows can easily be added to a cart via the table menu or simply by drag and drop onto the desired cart. Carts are rapidly synchronized between tabs within a browser session and user can share them in a collaborative manner using TBro’s controlled import and export functions.

Whole carts are visualized similar to individual unigenes or isoforms. Expression results are displayed as heat map for multiple selected conditions or tissues. Results from DE tests are graphed in a Bland–Altman plot [MA plot; (26)]. The latter is especially useful to localize selected unigenes or isoforms within the context of an entire expression experiment. Users can annotate Carts with an alias as well as a detailed description and store the cart itself and its corresponding annotation within TBro’s database. The OpenID-based user authentication system enables hundreds of users to store personal annotations generically however eliminating the need for its own centralized login system.

## Implementation

**Figure 1.**
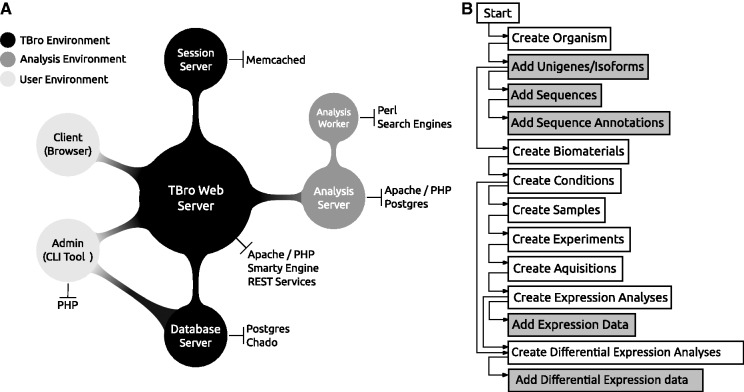
(A) TBro’s architecture is divided into three sections. The TBro environment builds the backbone with the central web server. The web server is connected to the database server and the session server for caching. The analysis environment is used to perform computationally intensive tasks. It is divided into a server and an arbitrary number of workers that can run on heterogeneous systems. The user environment consists of the client (a web browser) which is used to interact with a running instance of TBro and the command line tools which are used to import and manage data by a qualified administrator. (B) A typical data import hierarchically prepares and adds all transcriptomic data sets. Tasks performed by TBro-db are coloured in grey while tasks performed with TBro-import are coloured in white. The complete workflow tightly builds on the reference Chado schema to ease maintenance and usability.

TBro is divided into three environments (Figure 1A). The user environment (Figure 1A, light grey) consists of a client interface and an admin interface, which is used to control TBro. The admin tools are implemented in PHP with a command line interface (CLI) using multiple pear packages (Log, Console_CommandLine, Console_Table and Console_ProgressBar), propel for database abstraction (object-relational mapping), and phing for setting up databases and web interfaces. The client interface is structured using PHP and javascript with the Foundation Front-end framework. User interface interactions such as drag and drop capabilities, effects, widgets are built with the jQueryUI library. Displayed tables are created using the DataTables plugin for jQuery to make tables searchable and add multi-column ordering functionalities. Experimental as well as sequence annotation data are visualized using the CanvasXpress (25) graphing library. The Front-end is developed under the convention of the Document Object Model (DOM). DOM traversals, modifications and event binding are handled with jQuery. Ajax (Asynchronous JavaScript and XML) is used to update the parts of the frontend without reloading it completely. Data collections, arrays, and objects are manipulated using Underscore.js. Dynamic content is directly injected into the front-end using Underscore.js client-side templating.

TBro’s core environment (Figure 1a, black) consists of an Apache web server, delivering the web interface and providing core functionalities as atomic web services as well as a PostgreSQL server hosting the modified Generic Model Organism Database (GMOD) Chado database (27). Caching capability is provided by a memcached (28) server. The separated provision of each component provides high-availability and allows for resource optimizations (e.g. load balancing). REST Web services are written in PHP and return results formatted as JavaScript Object Notation (JSON). Database queries are logged and optimized using loggedPDO. Users are authenticated with lightOpenID. User session data is stored with webStorage on the client side to optimize server requests. Sequences and sequence annotations are stored using the Chado sequence module. Relationships between features such as unigenes and isoforms or proteins and protein domains are modelled using the feature relationship table. Quantification and DE testing results are stored in two newly introduced tables. Both tables complement the Chado Mage module to easily store non-microarray expression data. Future releases will store tabular data (e.g. quantification and DE testing results) using PostgreSQL NoSQL capabilities to speed up requests. User annotation data from carts and individual annotations are kept in a specifically created table (webuser_data). User data received from the front-end is inserted as decomposed binary format (JSONB).

The analysis environment (Figure 1A, dark grey) is used to perform computations like BLAST searches. Jobs are triggered by users via the web browser and tracked in a separate database. An arbitrary number of workers on heterogeneous host systems (currently Linux and Windows are supported) is utilized to run the job. Workers query the database for unallocated jobs, run them and report the results back to the database. The status of the job and eventually the results are accessible by the user via a unique URL. The analysis environment builds on a modular structure to easily extend it to other tools (e.g. HMMER for profile based searches).

## Usage

**Figure 2.**
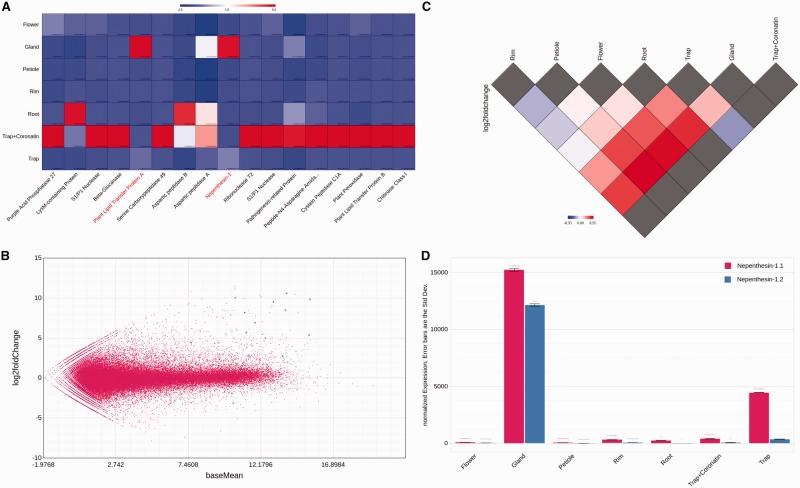
(A) Z-transformed expression heatmap of a cart containing putative members of the hydrolytic cocktails secreted by Venus flytrap during its hunting cycle. Two unigenes are being expressed in a non-stimulated gland specific manner. (B) MA plot for the same cart based on DE testing results from DESeq. The plot indicates that most members of the hydrolytic cocktail are being highly expressed compared to the majority of the unigenes. (C) Triangular visualization of the DE testing results for an individual gene (Nepenthesis-1). (D) Simple expression barplot of the Nepenthesin-1 gene with two isoforms showing different expression patterns. All plots were generated directly in TBro. Z-transformation, scaling and layouts were adjusted using functions from CanvasXpress context menu directly in the browser.

TBro knows two principal roles: administrator and user. The administrator imports and manages data using a CLI while the user accesses and searches the data with a web browser. The CLI is divided into three subcommands, TBro-db for managing data values (list, insert, edit and delete of e.g. contacts, organisms), TBro-import for importing multiple data values from files (e.g. ids, sequences) and TBro-tool which provides helper scripts (e.g. format converter). All tools come with support for auto completion in Linux environments. The CLI tools hierarchically prepare and import all data sets but can also be used to retrieve data from the database. An exemplary import workflow is available in the TBro documentation (http://tbro-tutorial.readthedocs.org). Sequence information and relations are imported by supplying relation maps (Unigene → Isoforms and Isoform → Open Reading Frame) and simple fasta files. The same is done for generic pathway associations (EC → KEGG Map). Annotation results are imported using a two-column tab-separated file (Sequence ID → GO/EC/Synonym) or source-defined multi-column files (Interpro, RepeatMasker, MapMan). Expression counts and DE results are imported after deep modelling the sample relations with TBro’s database control tool (TBro-db, Figure 2B). Each expression dataset is associated with a biomaterial (e.g. tissue), a condition (e.g. treatment) and a sample name (e.g. replicate-1) according to the Chado database schema. The combination of biomaterial, condition and sample name is connected with an experiment. Each experiment is assigned to one or multiple acquisitions corresponding to a sequencing runs or array hybridization. Acquisitions are associated with a corresponding analysis e.g. quantification and normalization of unigene and isoform counts or DE test results. Finally, the datasets are imported by simply supplying a quantification and analysis id.

The online demo (http://tbro.carnivorom.com) hosts data from the recently published Venus flytrap *(Dionaea muscipula)* deep transcriptome sequencing project (Bemm et al., 2016, in press). The unfiltered data sets contain 315 584 isoforms for 183 578 subordinate unigenes. A total of 3 221 001 annotation entries of various types are stored within TBro’s database backend. Expression data from four experiments with a total of 39 samples contain 19 467 318 distinct expression values and results for 2 744 423 DE comparisons are aggregated. The total size of the PostgreSQL database on disk is approximately 14 GB. All components of the Venus Flytrap TBro instance are running on a single virtual machine [Intel(R) Xeon(R) CPU E5-2640 v3, 2 cores, 8 GB RAM, Ubuntu 12.04, 64 bit].

One of the major questions during the deep transcriptome sequencing project of the Venus flytrap was about the nature and abundance of the hydrolytic enzymes which are secreted by specialized glands on the inner trap surface to digest animal prey. Several high-throughput proteomics experiments using different stimuli (insect and hormone treatment as well as mechanical stimulation) were conducted to stimulate secretion and detect hydrolytic enzymes in *Dionaea’s* digestive fluid. Following sampling of the secretion fluid, peptides were identified by mass spectrometry and mapped onto the reference transcriptome. Thereby 368 isoforms, respectively their deduced proteins, were identified as secreted independent of the nature of the stimulus. The resulting isoforms were searched within TBro and stored using its cart system. This initial ‘secretome’ cart was searched for entries exhibiting an annotated signal peptide (indicative for secreted proteins) employing the cart annotation search. Eligible isoforms were added to a ‘filtered secretome’ cart. Subordinate unigenes were added to the new ‘filtered secretome’ cart via the table menu and DE results from insect-stimulated glands (exp008) were visualized using a MA plot (Figure 2B). It became immediately obvious that the hydrolytic cocktail consists of enzymes being already expressed in non-stimulated glands (Figure 2B, blue dots with log_2_ fold change < 0, 2 unigenes) and those triggered upon insect stimulation (Figure 2B, blue dots with log_2_ fold change > 0, 15 unigenes). The two differentially expressed unigenes in non-stimulated tissues were further analysed with TBro’s triangular DE plot using an expression experiment comprising different non-stimulated tissues (exp001, Figure 2C). This plot revealed that the two unigenes, encoding Nepenthesin-1 and a Lipid Transfer Protein (LTP), are indeed excessively transcribed in a gland specific manner. The refined cart was directly used as supplementary data for the publication and to ease the review process.

Altogether, TBro successfully enhanced collaboration of numerous researchers working in the Venus flytrap transcriptome project team. It was particularly helpful to visualize expression strength or expression variability in publication-associated carts (Figure 2A). It was further intensively used to identify representative isoforms for individual unigenes using its adjustable expression bar plots (Figure 2D). Researchers frequently visualized DE test results using TBro’s triangular DE plot (Figure 2C) to identify DE patterns over a large set of different tissues. Finally, TBro’s pathway module was used to provide functional associations (e.g. Jasmonic acid biosynthesis, Supplementary Figure S2).

## Conclusion

TBro provides simple-to-use interfaces to (i) inspect and refine functional annotations, (ii) analyse and visualize expression as well as (iii) DE testing data. It handles user derived sets of unigenes/isoforms as well as entire experimental datasets and thus outperforms competing packages in terms of functionality, user-friendliness and flexibility. The cart system helps collecting, organizing and sharing biological meaningful sets of unigenes/isoforms and thus offers an effective way to export meta-data for external review. Building on the Chado database schema empowers TBro to handle complex representations of biological knowledge and a multitude of different data types. Although TBro was developed with RNA-seq experiments in mind, it can easily be adopted to host proteomic or other quantification data sets. Furthermore, it provides interoperability between different biological databases and applications of the GMOD toolkit. The modular backend, organized into different environments and the heavy use of highly flexible atomic services allow an easy extension of TBro’s functionalities in the future. It also provides a fast prototyping platform to test and develop functionalities for genome-centred data warehouse systems such as Intermine and Tripal. Upcoming releases will introduce cart operations such as union or intersection as well as transformations (e.g. unigene ↔ isoform) to further ease TBro’s usage. Finally, we aim to develop new features that enable users to switch between organisms or data releases in context of their personal carts again using Chado’s built-in relationship model.

## Availability

TBro is available as docker images (https://hub.docker.com/u/tbroteam) as well as source code (https://github.com/tbroteam). It is easily set-up using preconfigured docker images. Core applications, databases and job handlers are distributed in separate images. Functional tests are continuously performed with Travis-CI (https://travis-ci.org/TBroTeam/TBro) while code review is automatically performed by codeclimate (https://codeclimate.com/github/TBroTeam/TBro). A tutorial leads user through the installation as well as analysis process (https://tbro-tutorial.readthedocs.org). TBro is distributed under the MIT license. All included modules have compatible licenses (see Supplementary Table S1). The CanvasXpress (http://canvasxpress.org) release distributed with TBro is an earlier version available under the LGPL. Nevertheless, its version easily updated during the setup procedure.
